# Supplementary material for: Brain Fog and Fatigue following COVID-19 Infection: An Exploratory Study of Patient Experiences of Long COVID
Source: Int J Environ Res Public Health. 2022 Nov 23;19(23):15499. doi: 10.3390/ijerph192315499 (PMC9737348; doi:10.3390/ijerph192315499)
Supplement: Supplementary file 1 [file ijerph-19-15499-s001.zip › ijerph-2008883-supplementary.pdf]

Supplementary Table S1. Participants' descriptive and metaphorical language of the presentation of cognitive complaints ("brain fog") and fatigue in their own lives

Descriptions of Symptom Presentation	Illustrative Quotation(s)
Symptom: Cognitive complaints (Brain fog)	
**Head feeling fuzzy, foggy, or empty**	"…it feels empty in my head…"<br>"…you just kinda feel funny…you're not sharp"
**Memory issues (e.g., forgetfulness)**	"…I'm not very good at timelines right now."<br>"…I describe it as I'm speaking in a foreign language that I used to know."<br>"…the forgetfulness part, I reason it like I got Alzheimer's…"
**Issues with speaking and/or word recall**	"I'm mixing words, like the beginning of this word to the end of that word, but it just rolls off my tongue like it's a normal word."<br>"I was losing words…I would just be stuttering to get a word out…it was just lost, and I couldn't find it."<br>"…I would know what the word is, but I verbally couldn't get the word out."
**Difficulty listening and/or language processing**	"…my input and my output weren't speaking to each other."<br>"…when there's too much conversation…I can't disperse it. I just shut down."
**Visual processing**	"I know the next step to click on the computer and I'm just staring at it like it's a foreign language"<br>"…I get overwhelmed when I start reading though the material…they just feel like garble some days..."<br>"I get numbers turned around."
**Inability to focus or multitask**	"…it takes forever to work through different projects…"<br>"I couldn't think straight anymore."<br>"Just tryin' to think and process and things getting kind of muddied and jumbled up."
**Difficulty following steps in a sequence**	"I can't remember what the next click is on the computer"<br>"…my brain freezes and I can't figure out…in what order steps go."<br>"I would just do weird things…I would pour a bowl of milk and sit down and I'd forget my cereal"
**Losing time and/or "blanking"**	"…suddenly, I think back and I'm like, I'm not sure what I just did for the last two hours"<br>"I would just stop talking 'cause I didn't remember what I was talking about,"
**Difficulty planning or problem-solving**	"I get overwhelmed when I start reading through the material…[the instructions] just feel like garble some days…"<br>"…problem-solving is difficult for me lately…I take more time, usually. And sometimes I ask some employees for help."
**Mental fatigue**	"Some days I'm just so mentally drained…"<br>"…even just listening to all that noise takes a lot out of me."<br>"…the more I would try to think, the more the headache would get worse…"
**Feeling inadequate, incompetent, or unproductive**	"…my brain power is reduced and I'm havin' to focus on just basic essentials."<br>"…my brain does not work right now. I don't know what I need."<br>"…I can't trust myself going and doing things."
**Stress and/or anxiety**	"My anxiety level is higher. I get anxious easier than I used to."<br>"…I get stressed 'cause I can't do something."
Symptom: Fatigue	
**Mild tiredness**	"I feel kinda rundown"

	"…I was more tired than usual."
**Extreme exhaustion**	"…I cannot be awake, like I have to sleep immediately."<br>"…some days I'm just exhausted from the get-go."<br>"Just a feeling of your body being heavy and profound fatigue."
**Disproportionate response to physical exertion/Lack of stamina**	"I had a hard time making it up and down stairs…by the time I made it back down, I felt like I had used so much energy…"<br>"Mayo described it like a five-year-old iPhone battery. It may be charged up to 100 percent, but that doesn't mean full power. And even just a little bit of use drops it down to twenty quickly."<br>"I know I'm slow. I have a hard time keepin' up with my baby around the house."
**Lightheadedness and/or Dizziness**	"…my head feels like it's floating like a balloon"<br>"…my desk chair feels like it's tilting sideways…"<br>"A good day looks like getting up, feeling refreshed and clear and not dizzy…"<br>"I'll start getting really just to the point where I'm worrying about like falling. or something, and then I make sure to stop and rest."
**Weakness**	"…I feel very weak all the time."<br>"I don't have my strength."
**Shortness of breath**	"It wasn't far, but it was enough to wind me."<br>"Three blocks seems to be my max right now before I have to sit down and catch my breath."
**Lack of energy**	"I don't have the energy to get up and do anything."<br>"…I'd spend the entire weekend just trying to recover from a normal week."
**Lack of stability**	"…I would have difficulty even moving around the house without hanging on to whatever items were close by to give me stability and ability to stand."
**Sleep issues (e.g., insomnia)**	"…and sometimes I have crazy insomnia too"<br>"Sleep…last night was pretty rough. I had restless legs."
**Ineffectiveness of rest or sleep**	"So, I would wake up…and I really wouldn't feel refreshed or energized."<br>"It doesn't necessarily make me feel rested when I wake up, but it's like, I don't have any other choice."
**Altered senses**	"I'm still light sensitive—I work with my lights off now and just window light"<br>"I like it quiet…I like it dark. Light bothers."<br>"When I get really tired, I have the smell of gasoline that I just can't get out."
**Headache**	"I cannot exercise without any kind of post-exertion malaise headache starting up."<br>"We'll play one game, but I don't wanna do two games…because that'll make my head hurt."

Supplementary Table S2. Impact of PASC symptoms on different domains of daily life

Sphere	Illustrative Quotation
Impact on daily activities	
**Running a household**	I was always misplacing things. I couldn't find my shoes or – yeah and couldn't concentrate.
**Relapsing-remitting**	I will say probably the brain fog is on or off. I can have a really good week and I feel like, "Oh, this is good." I get some energy, so then I do a lot. Then it ends up being then the next week where I almost crash and hafta sleep so much just to recover. I'm trying to do better at pacing myself…
**Relapsing-remitting**	I was doing the dishes and my head just started nodding off because I was just so tired, so I put the dishes down and took a nap. I couldn't stay awake. It's gotten better, but I still have little waves where I have a day or two where just, the exhaustion is so much that I'm sleeping a lot…
**Unpredictability**	Until last week, every single day if I could take a nap, I would. I was exhausted. That's not like me…I love to cook, and I love to cook fancy meals. There's not much doin' that now…This week I was able to taste a couple of things, but that can go away again. Every time I think it's starting to come back and—it goes again.
**Unpredictability**	I think that you just can't plan on it because you never know when – I have good days, and I have bad days.
**Driving**	…when I come down to [the city] for appointments, I can't focus on the driving down there. There's too much goin' on. I always have to have a driver. I can drive around home here 'cause it's a little more rural. I'm used to it, and I can handle that.
Impact on work/employment	
**Employer support**	…I'd taken a leave of absence…that ended, and, actually, they let me still use more vacation and sick time that I had off. Finally, I ran out of that, and I was still not able to drive or be up and really functioning and so I resigned.
**Multi-tasking**	I can sit there and stare at something, which I would normally be able to just, boom, you know what I mean …
**Self-perception**	I was a person that worked forty to sixty hours a week [as a caregiver]…I take care of people. I took care of friends, and now, it's none of that.
**Self-perception**	I feel like I'm dropping the ball a lot. I don't like letting my students down and my staff. It's impacted my performance, it's impacted my self-perception of whether I do a good job or not, and that tends to crack in on my self-esteem…
**Colleague perceptions/stigma**	I think what it really comes down to, because it's invisible. When [colleagues] see you have a good week, it's like, "Oh, okay. She's back to her normal self." I am. I'm having a great week, but more, I just terrified of, okay, what am I gonna do that's going to push me into a crash again.
**Colleague perceptions/stigma**	But with work, it's interesting to see who reaches out to me and checks up on me and who doesn't…people that were on my team—I mean, they watched their [colleague] fall apart and a number of them have reached out or asked to come over and bring me lunch or just have a conversation…Then there are others that I wish their silence surprised me more.

**Unemployment/Loss of income/benefits**	They let me go…it left a bad taste in my mouth, but I recognize that I'm not particularly marketable as an employee if I were to go interview somewhere else, so I would need [former employer] as a place I can come back to and say, "I used to do these twenty things. Can I come back and try doing four of them, just to see if I can?" And have really, quite the accommodations made for me.
**Unemployment/Loss of income/benefits**	I'm on nothing. My big thing is, [the effect of] COVID for my income, is, I have none. I'm fighting that now…You just don't realize when something doesn't work right in your world—how much it really does affect you in certain things…
Impact on personal/social relationships	
**Loss/Change in roles**	Well, my older sister is now takin' full responsibility of taking care of my mother, who has Alzheimer's, 'cause I can't do it anymore. I used to be the one to fully take care of her in her home and take her to her doctor's appointments. Now my younger sister takes her to those.
**Effect on partner**	My husband's been great…Whatever I need, he just tries to get me.
**Effect on partner**	I wanna go interact with wifey before she goes to work, but she'll come upstairs and ask if I need anything and I hate that I didn't even make it down.
**Effect on parenting**	I don't remember things, like, "Well, I told you, Mom"… or "I told you this." I can write it down, but I'm not—I don't remember it.
**Effect on relationships outside the household**	…I can tell they don't really know how to act around someone who's sick, and people get uncomfortable around someone who's sick or someone who has a family member who's died. It's hard for people, and I try to be conscious of that.
**Effect on relationships outside the household**	…I think for them too, that's hard because on their end, they don't understand it because they've not dealt with long COVID symptoms…until you go through it, you just don't know.
**Feelings of isolation - Internal causes**	I can't be around a group of people. One on one, I'm not too bad now.
**Feelings of isolation – internal causes**	I just feel like I'm out of touch with the world, with the people I care about…
**Feelings of isolation – external causes/stigma**	…they're kinda quick to say, "Well, what did you have pre-existing before you had COVID?" As though it was kinda my fault…
**Feelings of isolation – external causes/stigma**	Yeah, there's a lotta my friends that I don't even have the patience to talk to anymore. I just got into a big argument with one of 'em about havin' vaccination…we're supposed to go on a [major] group trip…and I don't think I'll be doin' that.
**Stigma and difficulty being believed**	It bothers me to all of a sudden be looked at not like you're making it up, but maybe you're, I don't know—"Really, You can't taste anything?" It's just very frustrating. My immediate family who knows me well knows that that's not me. They know that's not something that somebody would fake.
**Changing the perceptions of others**	I was just told at a [celebration] a couple weeks ago that a friend of mine… [she] come over and said, "Hey, I got some people to get their COVID shots because they didn't know about you."
**Changing the perceptions of others**	There's a really, really horrible, long-term, middle-ground that is life-altering, and I'm living it…This is not in our head. This is not a lack of confidence. This is not an excuse to work from home. This is not a way to get attention. This is

	something that we don't understand why it's happening to us. There isn't—the doctors don't understand either. We're trying to get better, but if you could help us out by getting your goddamn shot and putting your goddamn mask on, that'd be great too.
